# Privacy protected text analysis in DataSHIELD

**DOI:** 10.23889/ijpds.v1i1.289

**Published:** 2017-04-19

**Authors:** Rebecca Wilson, Oliver Butters, Demetris Avraam, Andrew Turner, Paul Burton

**Affiliations:** University of Bristol

## Objectives

DataSHIELD (www.datashield.ac.uk) was born of the requirement in the biomedical and social sciences to co-analyse individual patient data (microdata) from different sources, without disclosing identity or sensitive information. Under DataSHIELD, raw data never leaves the data provider and no microdata or disclosive information can be seen by the researcher. The analysis is taken to the data - not the data to the analysis.

Text data can be very disclosive in the biomedical domain (patient records, GP letters etc). Similar, but different, issues are present in other domains - text could be copyrighted, or have a large IP value, making sharing impractical.

## Approach

By treating text in an analogous way to individual patient data we assessed if DataSHIELD could be adapted and implemented for text analysis, and circumvent the key obstacles that currently prevent it.

## Results

Using open digitised text data held by the British Library, a DataSHIELD proof-of-concept infrastructure and prototype DataSHIELD functions for free text analysis were developed.

## Conclusion

Whilst it is possible to analyse free text within a DataSHIELD infrastructure, the challenge is creating generalised and resilient anti-disclosure methods for free text analysis. There are a range of biomedical and health sciences applications for DataSHIELD methods of privacy protected analysis of free text including analysis of electronic health records and analysis of qualitative data e.g. from social media.

